# Integrative Brain Dynamics in Childhood Bullying Victimization: Cognitive and Emotional Convergence Associated With Stress Psychopathology

**DOI:** 10.3389/fnint.2022.782154

**Published:** 2022-04-27

**Authors:** Iryna S. Palamarchuk, Tracy Vaillancourt

**Affiliations:** Counselling Psychology, Faculty of Education, University of Ottawa, Ottawa, ON, Canada

**Keywords:** bullying victimization, cerebral functional activity, executive functions, memory, psychopathology, stress, theory of mind

## Abstract

Bullying victimization is a form of psychological stress that is associated with poor outcomes in the areas of mental health and learning. Although the emotional maladjustment and memory impairment following interpersonal stress are well documented, the mechanisms of complex cerebral dysfunctions have neither been outlined nor studied in depth in the context of childhood bullying victimization. As a contribution to the cross-disciplinary field of developmental psychology and neuroscience, we review the neuropathophysiology of early life stress, as well as general psychological stress to synthesize the data and clarify the versatile dynamics within neuronal networks linked to bullying victimization. The stress-induced neuropsychological cascade and associated cerebral networks with a focus on cognitive and emotional convergence are described. The main findings are that stress-evoked neuroendocrine reactivity relates to neuromodulation and limbic dysregulation that hinder emotion processing and executive functioning such as semantic cognition, cognitive flexibility, and learning. Developmental aspects and interacting neural mechanisms linked to distressed cognitive and emotional processing are pinpointed and potential theory-of-mind nuances in targets of bullying are presented. The results show that childhood stress psychopathology is associated with a complex interplay where the major role belongs to, but is not limited to, the amygdala, fusiform gyrus, insula, striatum, and prefrontal cortex. This interplay contributes to the sensitivity toward facial expressions, poor cognitive reasoning, and distress that affect behavioral modulation and emotion regulation. We integrate the data on major brain dynamics in stress neuroactivity that can be associated with childhood psychopathology to help inform future studies that are focused on the treatment and prevention of psychiatric disorders and learning problems in bullied children and adolescents.

## Introduction

Bullying victimization (BV) is a form of chronic psychological stress caused by interpersonal aggression that is repeatedly directed at a person who wields less power than their abuser (Olweus, 1994). Approximately one in 10 children worldwide are bullied regularly by their peers and another 30% of children are bullied on occasion (e.g., Nansel et al., 2001; National Academies of Sciences, Engineering, and Medicine, 2016; Biswas et al., 2020). Strong and growing evidence shows that BV is an intense psychological stress–targets of BV suffer chronic emotional distress which compromises their mental health and leads to persistent physical and social dysfunction, as well as poor academic achievement (e.g., Vaillancourt and Palamarchuk, 2021). The adverse correlates of BV similarly affect children worldwide (e.g., McDougall and Vaillancourt, 2015; Jantzer et al., 2021). Moreover, BV can lead to psychological adjustment problems and persistent mental health dysfunctions that extend well into adulthood (e.g., Arseneault et al., 2010; McDougall and Vaillancourt, 2015; van Geel et al., 2016; Moore et al., 2017; Vaillancourt and Palamarchuk, 2021).

Neurocognition plays a central role in the development of psychological stress. First, psychological stress is a physiological response to a salient stimulus that is perceived as a stressor such as a challenging event or behavior that disturbs the individual emotionally and thus triggers neurocognitive reactivity to adapt. Second, it is the cognitive appraisal that assigns the severity level to a stressor (i.e., interpretation of the stressor but not the nature of a stressor *per se*). If the stress is extreme and the stressor is uncontrollable, as is often the case with BV, neurocognitive stress reactivity can deviate toward maladaptation and psychopathology. In other words, the main effect of psychological stress is due to the perceived severity and controllability of the stressor, as well as the timing of the stressor (i.e., novelty/acuity/chronicity, Palamarchuk and Vaillancourt, 2021). That is why we hypothesize that the impact of BV on mental health will not be that different from the impact of other forms of psychological stress, such as childhood maltreatment, although we acknowledge that unique nuances may exist. We also recognize that the presence of physical/sexual abuse can aggravate psychological harm (e.g., Lereya et al., 2015); however, this interaction is beyond the scope of this review. Our reasoning for limiting our review to BV is because most neurobiological reviews have focused on child maltreatment and not on this other notable childhood stressor that affects far more children and youth worldwide (UNICEF, 2020).

Children are particularly vulnerable to the negative effects of psychological stress because the emotional and cognitive adjustment problems occur during a time when neuronal development is taking place (e.g., Sowell et al., 2004; Zhu et al., 2011; Cowell et al., 2015; Caballero et al., 2016). Emerging evidence supports that childhood BV has a profound effect on children’s brain development. For instance, Du Plessis et al. (2019) found longitudinal links between childhood BV and structural changes in the right ventrolateral (vl) prefrontal cortex (PFC) moderated by cortisol levels in adolescent boys but not adolescent girls. High BV with low daily cortisol output and/or a steeper diurnal slope was associated with reductions in the right vlPFC surface area, and high BV with high daily cortisol output and/or a low flatter diurnal slope was associated with a larger right vlPFC surface area. These findings highlight the differential effects of BV on brain development. In particular, decreased cortisol levels in bullied adolescents might indicate stress adaptation because reduced surface area and increased cortical thinning of the PFC during adolescence are part of normal development. Quinlan et al. (2020) provided evidence that BV at age 14 was indirectly associated with generalized anxiety *via* reductions in volume of the left dorsal striatum (nucleus caudate and putamen) at age 19. Muetzel et al. (2019) found that 10-year-old targets of BV had increased cortical thickness in the left fusiform gyrus, yet the findings might relate to either the pre-existing differences or to the consequences of BV. Although it is important to compare the influence of BV across the lifespan, such as childhood vs. adulthood, we emphasize the integrative and inter-disciplinary research on childhood BV because the adult literature is limited. We predict that adulthood BV would differ from childhood BV mainly due to the distinct factors that hinder stress appraisal and increase stress sensitivity. Namely, the etiology of childhood BV sensitivity relates to the neurocognitive immaturity that contributes to the amygdala—PFC networking specifics, which are reviewed. Adulthood BV sensitivity likely relates to the frontrostriatal loop engaged in motivation, habits, reward-learning, and decision-making (for the nuances in general psychological stress, see Palamarchuk and Vaillancourt, 2021).

The primary focus of this review is to highlight childhood BV and psychopathology through the lens of neurocognitive functioning. The novelty is that we present BV research on executive functioning by outlining multiple levels of cognition including learning, semantic cognition, cognitive flexibility, and processing of social and emotional information that involves behavioral regulation (i.e., social cognition, Shany-Ur and Rankin, 2014). We offer an innovative approach to the topic of BV. Specifically, an integrative brain dynamics model of cognitive and emotional convergence is presented based on the neurophysiological evidence of the effects of early life and general psychological stress with the aim of helping explain why BV is likely so robustly linked to psychopathology. We also discuss the application of the results and provide examples to help with the design of future studies that are focused on the treatment and prevention of psychiatric disorders and learning problems in bullied children and adolescents. It is our cross-disciplinary contribution to cognitive neuroscience, social neuroscience, developmental neuropsychology, psychological pedagogy, and psychiatry interface, which distinguishes the innovation of this review.

### Stress-Induced Neuropsychological Cascade

#### Neuroendocrine Reactivity Related to Psychological Neuromodulation

Psychological stressors are salient stimuli, such as events and behavior, that can trigger aversive emotions and feelings. When a negative value is assigned to the salient stimuli (cognitive appraisal), psychological stress occurs. Psychological stress is a physiological response governed by the neurocognition to stay focused on the challenge and adapt. This stress response does not principally relate to the stimulus *per se*, but to the perceived stress severity and controllability that depends on the stressor’s acuity (i.e., novel/unpredicted vs. homotypic/chronic). That is, the major difference between the stressor’s influence on neuromodulation is not due to the nature of the stressor (e.g., childhood maltreatment vs. BV) but rather due to the interpretation of the stressor (see detailed review by Palamarchuk and Vaillancourt, 2021).

BV is a psychologically stressful experience for children (Vaillancourt and McDougall, 2013) and the stressor experienced is far too often extreme and/or prolonged. In general, psychological stress initially activates the hypothalamic–pituitary–adrenal (HPA) axis that alters glucocorticoid levels (i.e., circulated cortisol concentration), an effect seen in bullied children (e.g., Ouellet-Morin et al., 2011a, b, 2013, 2021; Vaillancourt et al., 2011; Kliewer et al., 2019). Human and animal studies show that increased cortisol levels in response to stress can over-activate the glucocorticoid receptors that prevail in the hippocampus and PFC, areas of the brain primarily associated with memory and learning. This activation can trigger neurocognitive responses linked to memory, mood, behavior, and executive functions, which should theoretically help the individual cope with the stressor (e.g., De Kloet et al., 1998, 2018; De Kloet and Derijk, 2004; Barsegyan et al., 2010; Vogel and Schwabe, 2016; Palamarchuk and Vaillancourt, 2021). Indeed, childhood adversity does not exclusively harm cognitive functions; it can also promote developmental adaptation to resilience *via* the improvement of some cognitive functions (Ellis et al., 2017). Nevertheless, stress-associated outcomes differ according to the intensity/severity and controllability of the stressor, which relates to cognitive appraisal (aforementioned stressor’s interpretation), as well personal and environmental factors (e.g., Schneiderman et al., 2005; Palamarchuk and Vaillancourt, 2021). When a stressor is beyond the coping ability of the individual, impairment in cognition and metacognition (i.e., awareness of one’s own thinking/learning) can occur (e.g., Lupien et al., 2005; De Kloet et al., 2016, 2018; Palamarchuk and Vaillancourt, 2021), which is often the case with BV (e.g., Ouellet-Morin et al., 2011b; Sinclair et al., 2012; Vaillancourt et al., 2013; Liu et al., 2016; Carroll et al., 2019; Gini et al., 2019). For example, there is mounting evidence documenting that bullied children and adolescents struggle academically in comparison to their non-bullied peers (Nakamoto and Schwartz, 2010; Espelage et al., 2013). Yet despite this well-replicated literature, the prevailing view regarding the association between BV and academic achievement does not account for a neurobiological mechanism (see exception, Vaillancourt et al., 2013; Vaillancourt and Palamarchuk, 2021). Rather, this association is typically attributed to poor school attendance or poor mental health mediating the link between BV and academic achievement.

The impaired neurocognitive functioning found in bullied children might also emerge from altered cortisol concentrations (e.g., Vaillancourt et al., 2008, 2011; Ouellet-Morin et al., 2011b, 2013, 2021; Östberg et al., 2018b; Palamarchuk and Vaillancourt, 2021), that in turn, affect neurochemical mechanisms (i.e., neurotransmission) of memory, mood, and behavior. Specifically, stress can impact dopamine signaling in the PFC-striatum circuits (e.g., Gamo et al., 2015; Reneaux and Gupta, 2018), which plays an important part in neuromodulation (i.e., the capacity of a single neuron to regulate wide-ranging populations of other neurons), reward-motivated behavior, reinforcement learning (e.g., Ikemoto, 2007), and representational mentalizing (i.e., theory of mind) development (Lackner et al., 2010).

In fact, the alteration of dopamine signaling is linked to childhood adversity (e.g., De Bellis et al., 1994; Oswald et al., 2014; Egerton et al., 2016; Bloomfield et al., 2019). Dopamine signaling can be moderated by environmental and genetic factors in adolescence, a developmental period that is particularly vulnerable to the effects of stress on the PFC’s functions due to increased dopaminergic projections (Arnsten and Shansky, 2004; Oswald et al., 2014; Egerton et al., 2016). Polymorphism of the dopamine D4 receptor (DRD4) gene (i.e., shorter vs. longer alleles) was found to correlate with better representational mentalizing (Lackner et al., 2012), executive functioning, and social/emotional development in preschoolers (Pappa et al., 2015). Kretschmer et al. (2013) examined whether peer effects (negative vs. positive) on adolescent development were genetically moderated *via* the DRD4 polymorphism [the 7-repeat (7R) allele vs. the 4R allele]. Results indicated that the 4R allele was linked to higher susceptibility for the effects of BV and social well-being on later delinquency. Janssens et al. (2015) showed that peer rejection, a strong correlate of BV (Knack et al., 2012), was associated with rule-breaking behavior moderated by the dopamine transporter genotype (DAT1) in adolescents; in particular, the 10R-allele carriers showed more rule-breaking behavior in the context of high peer rejection, but less rule-breaking behavior in the context of low peer rejection. Cao et al. (2017) showed that functional polymorphism (TaqIA) in the dopamine receptor D2 (DRD2) gene (A2A2 genotype, i.e., no A1 allele) at age ~12 years predicted higher levels of depression at age 14 years in bullied adolescent boys, but not bullied girls. In contrast, among adolescent offenders, carriers of the A1 allele were more likely to be severely bullied compared to non-carriers of the A1 allele (Vaske et al., 2011). Thus, altered dopamine signaling may be a predisposition to BV and its pervasive psychopathological impacts, especially in adolescents who have poor executive functions, depression, and genetic risk factors.

#### Limbic Dysregulation and Emotion Processing

Besides the HPA axis, the principal stress response involves the locus coeruleus-norepinephrine system which relates to arousal, as well as fear supported by hyperactivation of the amygdala. This response has cognitive and behavioral effects, including arousal, attention, and cognitive flexibility (e.g., Skosnik et al., 2000; Morilak et al., 2005; Alexander et al., 2007; Valentino and Van Bockstaele, 2008). Although these effects can support careful assessment and strategic planning in unpredictable situations, they can also increase anxiety *via* the behavioral inhibition system associated with the lateral amygdala and the ventromedial (vm) PFC (e.g., Markett et al., 2018; Palamarchuk and Vaillancourt, 2021). In other words, behavioral inhibition ensues which involves the withdrawal from the danger, a reaction that is adaptive in this specific context. However, the continuation of inhibitory behavior is likely why we see the development of mental health problems including anxiety, depression, psychosis, psychosomatic, and eating disorders among bullied children (e.g., McDougall and Vaillancourt, 2015). For example, Weems et al. (2003) found that hyperarousal symptoms in adolescents with PTSD predicted the development of emotional numbing, which presumably relates to cognitive alterations in PTSD (see also Hayes et al., 2012; Palamarchuk and Vaillancourt, 2021).

BV is related to disturbed emotional processing and activation of the cerebral networks of social pain and monitoring (Rudolph et al., 2016; Will et al., 2016; Telzer et al., 2018, 2020; McIver et al., 2019). Within the cerebral networks of emotional processing, the essential role belongs to the amygdala, which if dysregulated, can contribute to psychiatric disorders (e.g., Monk et al., 2008; Brotman et al., 2014; Wegbreit et al., 2014). In bullied children, the amygdalar dysregulation can be seen in hyper responses to social exclusion (McIver et al., 2019), fearful faces (Swartz et al., 2019), and unexpected positive peer evaluation, in which high wariness in early childhood correlates with the severity of adolescent social anxiety (Jarcho et al., 2019). Moreover, amygdalar hyperactivity relates to the severity of BV and internalizing/externalizing symptoms, and to poor social self-esteem in bullied adolescent girls (Telzer et al., 2020). The neuronal mechanisms of anxiety are that repeated exposure to BV can facilitate the maintenance of extremely vivid aversive memories and evoke strong negative emotional responses such as acoustic perceptions or body sensations (Sansen et al., 2015). The traumatic memories can be intrusive as their activation can be triggered by even benign cues that remind the individual of the aversive event *via* an associative information network; this memory network can contribute to the development and maintenance of psychiatric dysfunctions such as social anxiety disorder (Iffland et al., 2014). Moreover, general anxiety levels have been shown to mediate a poor stress response in bullied children such as the negative relation between BV and cortisol levels during school lunchtime, a period of anticipated exposure to BV (Carney et al., 2010); whereas blunted cortisol responses to stress were found to be linked to increased social/behavioral problems in bullied adolescents (Ouellet-Morin et al., 2011b; 2021).

Stress-associated amygdalar dysfunction relates (but not exclusively) to the midbrain raphe nuclei and serotonergic (5-HT) pathways implicated in the downregulation of fear and anxiety (e.g., Bocchio et al., 2016; Palamarchuk and Vaillancourt, 2021) and in the enhanced active coping with inescapable stress in rodents in a time-locked manner (Nishitani et al., 2019). Uncontrollable stress is associated with the dysregulated dorsal raphe nuclei that affects 5-HT signaling (e.g., Amat et al., 2005), and alterations are linked to fear memory formation and retrieval (Sengupta and Holmes, 2019), as well as a higher risk for depression (e.g., Amat et al., 2005; Bocchio et al., 2016), greater susceptibility to future stress, and stress-induced anhedonia (Prakash et al., 2020). These serotonergic pathways can further compromise the physiology of various neuropsychological domains (e.g., Berger et al., 2009), including but not limited to those associated with BV— namely aggression (Bettencourt et al., 2013; Krygsman and Vaillancour, 2019), perception (Cole et al., 2014; Lavell et al., 2018; Östberg et al., 2018a), reward (Casement et al., 2014; Rappaport et al., 2019), attention/memory (Vaillancourt et al., 2011), appetite (Lee and Vaillancourt, 2018), and sleep (van Geel et al., 2016), with sleep problems mediating the link between delinquency and drug use in bullied girls (Sosnowski et al., 2016). In contrast, when a stressor is controllable, the vmPFC can inhibit the dorsal raphe nucleus’ stress-response, which in turn prevents learned helplessness and behavioral depression (e.g., Amat et al., 2005; Maier et al., 2006). In other words, the vmPFC plays a major role in coping with stress (i.e., strengthening of the escape response) and its deficient inhibitory control over the limbic circuits triggered by aversive events determines the uncontrollability of the stressor that affects behavior. BV can be an uncontrollable stress per its association with behavioral /psychological maladjustments in childhood (Arseneault et al., 2010; McDougall and Vaillancourt, 2015; van Geel et al., 2016; Moore et al., 2017; Vaillancourt and Palamarchuk, 2021) and interpersonal needs-hopelessness sequela that can indirectly moderate suicidal ideation in adolescents (Shin et al., 2016; also see Hong et al., 2015; Silberg et al., 2016). Hopelessness results from anticipating more negative than positive events, unachievable goals, and surrender (Marchetti, 2019; see also Palamarchuk and Vaillancourt, 2021); its high levels correlate with increased inflammatory reactivity to social stress in bullied adolescents (Giletta et al., 2018). This is a psychological defeat with an alarming global effect. For instance, BV is a high-risk factor of adolescent suicide attempts (Koyanagi et al., 2019), which is the leading cause of death among youth in high-income countries, accounting for 17.6% of all deaths (UNICEF).

### Neurocognitive Effects

BV exposure has been linked to significant memory impairment in children (Vaillancourt et al., 2011). Memory impairments are driven by glucocorticoid activation (Sauro et al., 2003) and cortisol-induced genomic mechanism that develops with the stress-activated translocation of glucocorticoid receptors to the neuronal nucleus (Kino et al., 2009; Argentieri et al., 2017). Epigenetic mechanisms associated with stress primarily belong to the level of brain-specific DNA methylation, which leads to stable gene repression (e.g., Brenet et al., 2011) and has a distinguished impact in early life (e.g., Lubin et al., 2008; Szyf and Bick, 2013; Alexander et al., 2018).

Exposure to severe and/or chronic stressors can alter glucocorticoid signaling, resulting in dendritic atrophy and reduced spine density that affects synaptic plasticity (McEwen, 1999, 2000, 2001; Sousa et al., 2000; Moench and Wellman, 2015; Madalena and Lerch, 2017; Urban et al., 2019). Spine density is necessary for neuronal network formation, an essential component of cognition, including memory and learning that is promoted in the PFC and hippocampus (Amat et al., 2005; Gilabert-Juan et al., 2013). Stress can further induce cerebral morphological changes; for instance, early life adversity is associated with volume reductions in the amygdala, medial PFC, and hippocampus (in exposure to threat) and frontoparietal regions (in children exposure to deprivation; Kim et al., 2019; McLaughlin et al., 2019), as well altered white matter tracts (Kim et al., 2019), that increase the risk of developing PTSD (Yehuda, 2009) and neurodegeneration (Vyas et al., 2016). The alteration of cerebral morphology is moderated by interactions between childhood adversity and genes, such as the S allele of *5-HTTLPR* (for the hippocampus), Met allele of *BDNF* (for the amygdala, hippocampus, PFC, and rostral ACC), and *FKBP5* gene rs160780 (for white matter), which have links to depression (Kim et al., 2019). Consistent with this idea, several researchers have found links between BV and symptoms of depression and PTSD in children who are targeted by their peers (Vaillancourt et al., 2011; Idsoe et al., 2012, 2021; Nielsen et al., 2015; Lee and Vaillancourt, 2018). And, as is the case with the association between poor academic achievement and BV mentioned previously, the association between PTSD and BV likely has a strong neurobiological underpinning. We hypothesize that the PTSD and depression symptoms found in some bullied children are in fact due to neurophysiological changes that are like the ones found in maltreated children, resulting from suppressed neurogenesis, stress-associated delayed myelination, as well as distorted apoptosis (Kavanaugh et al., 2017).

Neurogenesis refers to neuronal growth and development, where key events occur in dendrites and axons when neurons differentiate and mature. In dendrites, it is seen in the morphological changes such as increases in size and branching that are necessary for signal flow between neurons. In axons, the changes involve growth cones that interact with cell adhesion molecules to anchor the tissue substrate, which are required components for primary outgoing pathways. Neurogenesis contributes to synaptogenesis and cortical thickness. Its suppression affects the amount and type of signals the neuron receives (Belsky and de Haan, 2011), whereas BV relates to the cortical thickness changes in adolescents (Muetzel et al., 2019). In a multicohort analysis of the community-based data (Brazil, Canada, and Europe), Parker et al. (2020) showed the variance in cortical thickness during maturation was linked to dendrite, spine, and myelin genes, which are also enriched in genes associated with psychopathology.

Myelination is the coating process of the axons that increases the speed of nerve transmission up to 100 times. Its delay has a significant impact on neuronal circuits’ functioning (Belsky and de Haan, 2011). Delayed myelination can be detected by reduced fractional anisotropy in diffusion tensor imaging. In fact, maltreated children with PTSD present with reduced fractional anisotropy in the corpus callosum, the primary white matter tract with interhemispheric projections necessary for the processing of emotional stimuli and memory functions (Jackowski et al., 2008). Apoptosis is physiologically programmed cellular death, which is elevated during psychological stress due to neurochemical changes. In particular, animal studies suggest that severe stress causes neuronal apoptosis in the hippocampus related to the neurotransmitter imbalance (between glutamate and GABA), which may explain the findings pertaining to PTSD (i.e., inhibition of the HPA axis activation, anxiety, and poor learning; Gao et al., 2014). Although not directly assessed in bullied children, we hypothesize similar neurobiological findings will be obtained, because, like child maltreatment, BV is a form of interpersonal trauma (Idsoe et al., 2021).

From a therapeutic standpoint, we foresee that some neurocognitive dysfunctions may be reversible to a certain degree if the stressor is terminated (Conrad et al., 1999) and the neural harm is not related to a permanent loss of cells but rather is related to atrophy (McEwen, 1999, 2000; Sousa et al., 2000). The prediction is supported by the fact that while myelin plasticity substantially responds to chronic psychosocial stress (Laine et al., 2018), glucocorticoid treatment can initiate and enhance post-traumatic myelin formation, which is a cellular marker of cerebral regeneration (Chan et al., 1998). At the same time, glucocorticoid-related biomarkers (e.g., bedtime salivary cortisol and plasma dehydroepiandrosterone/cortisol ratio) are associated with the treatment of PTSD symptoms, including anxiety and depression in combat veterans (Yehuda et al., 2014).

#### Hippocampus-Dependent Learning

The neural factors of BV likely involve a complex interplay between hyperarousal, fear conditioning, and memory processing (i.e., learning and executive functions). Memory processing can be mapped onto the anterior cerebral network between the PFC, amygdala, hippocampus, and its adjacent cortex, the parahippocampal gyrus with peri- and ento-rhinal cortical areas (e.g., Raslau et al., 2014, 2015). In this anatomical network, the stress-response of the ventral hippocampus is distressed by the amygdala’s projections that are linked to fear conditioning and affective processes (e.g., Anagnostaras et al., 2002; Cenquizca and Swanson, 2007). The amygdala plays a critical role in stress-induced memory impairment *via* fear conditioning and explicit/associative memory alteration (e.g., McGaugh, 2004; Rabinak and Maren, 2008; Robbins et al., 2008) that can alter hippocampus-dependent episodic memory when coupled with a stimulus that is perceived as emotionally evocative (e.g., Tulving and Markowitsch, 1998; Whitlock et al., 2006). Generally, psychological stress *via* fear activates the amygdala, which impacts proper hippocampal function that is needed for encoding and working memory; severe stress can also lead to hippocampal atrophy and memory deficits (e.g., Bremner and Narayan, 1998). This stress-induced effect on hippocampus-dependent learning potentially contributes to the memory deficits found in bullied children (Vaillancourt et al., 2011; Sansen et al., 2015). Lee et al. (2018) showed that being verbally abused by peers in adolescence is associated with volume reductions in the left hippocampal subfields, a finding also documented with childhood maltreatment, another form of interpersonal trauma (Teicher et al., 2012; Riem et al., 2015). Emotional childhood neglect is linked to left hippocampal white matter reductions in adult patients with major depression (Frodl et al., 2010). As well, PTSD predicts right hippocampal volumes reductions (Carrion et al., 2007). PTSD symptoms inversely correlate with hippocampal and amygdalar (left) volumes in maltreated youth with PTSD compared to those without PTSD (Morey et al., 2016), whereas reduced hippocampal and amygdalar volumes partially mediate the relation between early life stress and behavioral problems (Hanson et al., 2015). Additionally, stress-induced hyperactivation of hippocampal glucocorticoid receptors suppresses neurotrophic factor and leads to atrophic changes in the dentate gyrus (a cortical region of the hippocampal formation; Amaral et al., 2007) that impairs long-term memory and promotes depressive symptoms in chronically stressed individuals (Malberg et al., 2000; Bekinschtein et al., 2008; Surget et al., 2011; Taliaz et al., 2011). Although these effects are well documented, more work is needed to clarify specifics of the brain structure and functioning linked to poor memory in bullied children and youth.

#### Striatum-Dependent Learning

Under stress, the amygdala also promotes functional connectivity with the dorsal striatum that reduces its functional connectivity with the hippocampus, impacting the quantity and quality of memory; in particular, the established coupling shifts memory encoding and retrieval from hippocampus-dependent (“cognitive”) to striatum-dependent (“habit”) mode (Packard, 2009; Vogel and Schwabe, 2016; Vogel et al., 2017; Zerbes et al., 2020). The dorsal striatum relates to the automatic/habit responses and attention regulation *via* the PFC and parietal cortical interactions (e.g., Lago et al., 2017) and the implication of this shift is that the memory process reduces flexibility and thus becomes more reflexive. Consequently, stress-induced poor attentional set-shifting (i.e., cognitive inflexibility), which is related to the medial PFC’s dysfunction (e.g., Floresco et al., 2008; Butts et al., 2013; George et al., 2015), results in behavioral inflexibility that has been observed in bullied children. Specifically, the individual relies on old ways of responding, which might not be adaptive or appropriate because they are linked to threats from the past and not the present.

Although studies are needed to clarify the links between BV and striatum-related cognitive deficit, Telzer et al. (2020) showed that severe BV was related to hyperactivation in the amygdala, ventral striatum, fusiform gyrus, and temporoparietal junction, which was associated with increased internalizing and externalizing symptoms in adolescent girls. Of note, the ventral striatum is preferentially engaged in emotional processes and its dysregulation can lead to anxiety (Lago et al., 2017). The link between BV and anxiety is well noted in the literature and supported by meta-analytic findings (Reijntjes et al., 2010; Wu et al., 2015; Moore et al., 2017) and longitudinal studies (Takizawa et al., 2014; Sentse et al., 2017; Drazdowski et al., 2021). Silk et al. (2014) also has shown that reactivity of the ventral striatum (nucleus accumbens) and insula is moderated by depression in adolescence. Lee et al.’s (2020) findings are that BV has an indirect effect on adolescent depression *via* volume increases in the nucleus accumbens.

Developmental sensitization to stress differentiates children at risk for anxiety symptoms and internalizing disorders with profiles of sustained cortisol elevation (i.e., morning cortisol and during acute stress exposure; Laurent et al., 2015). Specifically, high levels of cortisol’s response to stress are distinguished by anxiety and linked to the dorsal striatum (putamen) volume reduction during a sensitive period in neuroanatomical development in childhood. Of relevance, recall that Quinlan et al. (2020) showed that chronic BV was indirectly linked to generalized anxiety *via* reductions in the left dorsal striatum in adolescence. Egerton et al. (2016) found that childhood adversity was linked to increased striatal dopamine activity in adulthood. Oswald et al. (2014) showed positive associations between childhood trauma and current levels of perceived stress, which related to higher ventral striatal dopamine responses to amphetamine. Accordingly, difficulties in learning and adjustments found in BV targets (Nishina and Parra, 2019; Vaillancourt et al., 2011, 2013) could be related to the striatal-dependent shift in memory processing associated with cognitive inflexibility and anxiety, especially in adolescence.

#### Executive Functioning: Cognitive Flexibility

Executive functioning is a cognitive control based on the analysis of environmental information and processed sensory information to assist with information encoding and cognitive flexibility (e.g., rapid attention/task-shifting and proper behavioral adjustments), which is crucial for decision making and problem-solving in adaption to the challenges presented (e.g., De Kloet et al., 1998; Dajani and Uddin, 2015; Palamarchuk and Vaillancourt, 2021). This type of cognitive control relates the dorsal and vlPFC functioning (e.g., Wager and Smith, 2003; Barbey et al., 2013), that is linked to developmental increases in the frontoparietal and parietal-dorsal ACC functional connectivity in the right hemisphere, to a stronger degree in girls compared to boys (Langeslag et al., 2013). Stress can alter executive functions, which is seen in the aforementioned cognitive inflexibility such as poor task/set-switching ability linked to the medial PFC dysfunction (Floresco et al., 2008; Butts et al., 2013; George et al., 2015) that correlates to cortisol response (e.g., Plessow et al., 2012; Goldfarb et al., 2017). Cognitive inflexibility is related to binary decision-making, which is a defensive cognitive strategy to ergonomically conquer the social challenge by integrating insecurity predictability, reasoning, learning fortification, and monitoring behavioral strategies (Collins and Koechlin, 2012). Nevertheless, poor cognitive flexibility is associated with poor mental health, for instance, depression (Gotlib and Joormann, 2010; Murphy et al., 2012; Hou et al., 2016) and BV. For example, Medeiros et al. (2016) showed that 10- and 11-year-old targets of BV had lower cognitive flexibility that was associated with poor “cold” executive functions (related to the logical-rational part of the cognition) compared to perpetrators of bullying. In another study, Jenkins and Canivez (2021) found a negative association between BV and executive functions, including cognitive flexibility, self-monitoring, emotion regulation, inhibition, and initiation in 6th Grade to 8th Grade. McQuade (2017) demonstrated that executive functions moderated the relation between BV and increases in aggression over a year in adolescents.

The neurobiological mechanisms are that psychological distress might modulate an individual’s response to stress, whereas poor stressor controllability (capacity of the PFC, discussed above) relates to exacerbated fear and anxiety that affects cerebral networks of the executive functions seen in behavioral problems (e.g., Maier et al., 2006; Maier and Watkins, 2010). For example, this mechanism can be seen in the hippocampal-related approach—avoidance conflict in young healthy adults (O’Neil et al., 2015), amygdala-related greater risk-taking behavior in bullied adolescent girls (Telzer et al., 2018), and learned helplessness and behavioral depression related to the medial PFC in rodent models (Maier et al., 2006). In childhood, there are four common behavioral responses to stress: impulsivity (i.e., acting-out or overreactive), passive-aggressiveness (i.e., overly compliant or uncooperative), dependency (i.e., passive or demanding), and repression (i.e., withdrawn or anxious; Chandler and Shermis, 1986). These responses are commonly expressed in bullied children (Reijntjes et al., 2010, 2011), which we predict are likely shaped by altered executive functioning seen in a stress-induced narrowed attention that enhances binary discrimination where the focus is placed on the aversive details (i.e., attentional bias; Cohen et al., 2009; Byrom and Murphy, 2016; Otgaar et al., 2017; Palamarchuk and Vaillancourt, 2021). Relatedly, BV is associated with goal-oriented selective attention (Carroll et al., 2019) and greater negative appraisals of peers (Troop-Gordon and Ladd, 2005).

A potential contributor to the behavioral maladjustment of bullied children is stress-induced right amygdalar hyperactivity that alters ascending projections toward the PFC. This signaling can modify executive functions related to decision making in unpredictable conditions (Maier et al., 2006; Maier and Watkins, 2010; Gupta et al., 2011; O’Neil et al., 2015), which are governed by the dorsal/lateral PFC (e.g., Wager and Smith, 2003; Barbey et al., 2013). Adolescence is a particularly vulnerable period for the development of executive dysfunctions because of the peculiarities in brain development seen in the functional organization of cognitive networks (especially in the right hemisphere; Sowell et al., 2004; Zhu et al., 2011) that parallel with attentional shifting, working memory, response inhibition, and goal-directed behavior (Luna and Sweeney, 2004; Paus, 2005; Bunge and Wright, 2007; Yurgelun-Todd, 2007; Schmithorst and Yuan, 2010). For instance, white matter microstructure was found to be correlated with the impulsive behavior in adolescents, with sex-specific differences (Silveri et al., 2006). The consequence is that learning strategies and decision-making are suboptimal at a time when an individual needs full functioning of their faculties to deal with the toxic social stressor. Relatedly, Herringa et al. (2016) showed that childhood adversity predicted hyper responses to aversive images in adolescence seen in the right amygdala hyperactivity and increased the right amygdala → bilateral PFC (BA 9, 10), bilateral hippocampi → bilateral PFC (BA 8, 9), and right hippocampus → left ACC (BA 32) functional connectivity. Moreover, right hippocampus reactivity was negatively associated with internalizing problems, while childhood adversity predicted increased right amygdala-dorsal PF connectivity specifically for negative emotional stimuli for adolescents only with lower levels of internalizing problems. This could be related to the higher ability of the PFC to regulate amygdalar responses (i.e., the PFC → amygdala inhibitory control); yet more research is needed to clarify these findings.

**Figure 1:**
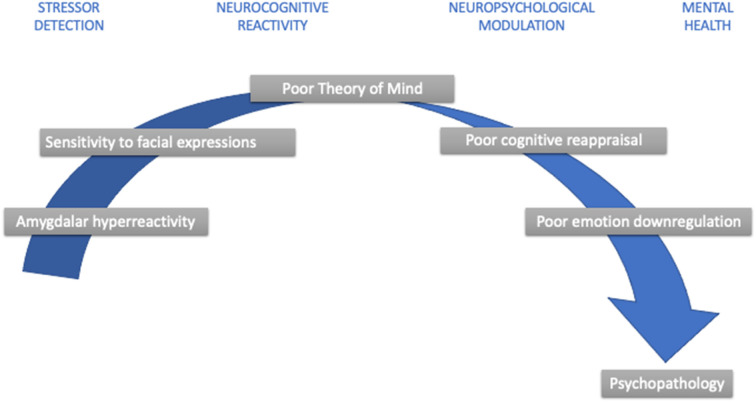
Developmental aspects in childhood stress psychopathology. Note: This simplified diagram summarizes developmental nuances that contribute to childhood stress sensitivity and increase psychopathology risk.

**Figure 2:**
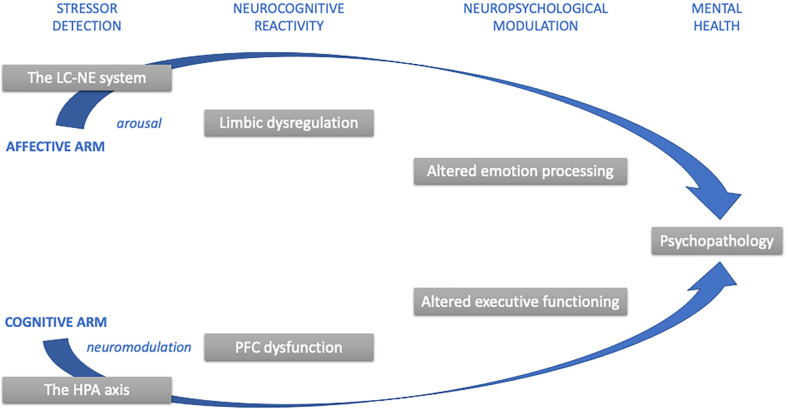
Cognitive and emotional convergence associated with stress psychopathology. Note: This simplified diagram summarizes the pathways of cognitive and emotional convergence in stress that can lead to psychopathology. *Abbreviations*: HPA, hypothalamic–pituitary–adrenal; LC-NE, locus coeruleus.

**Figure 3:**
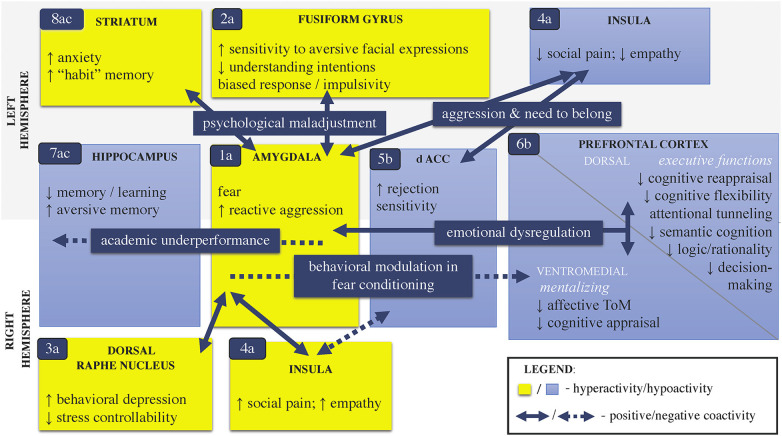
Major brain circuits associated with childhood stress psychopathology. Note: This schematic model integrates the major cerebral circuits (1–8) of cognitive and emotional convergence in stress neuroactivity that can be associated with childhood psychopathology. The model is a simplified syntax of the evidence in: [a] general psychological stress; [b] childhood BV; and [c] early life adversity. [a] The brain dynamics stem from (1) a hyperactivated amygdala following fear conditioning, which is a common neural response to an acute psychological stressor (i.e., “alarm-to-threat” stage, Palamarchuk and Vaillancourt, 2021). The magnitude of the amygdalar stress-reactivity can be mediated by the activity of the (2) fusiform gyrus, (3) dorsal raphe nucleus, and (4) anterior insula. [b] Hypothetically, the amygdalar overactivity can suppress the (5) dorsal/rostral ACC, which further can lead to (6) hypofunctioning of the vmPFC and thus impair mentalizing in young targets of BV. [a,c] Generally, in intense/chronic psychological stress, the amygdalar hyperactivity can (7) alter hippocampal memory encoding and (8) promote striatum-dependent memorization, which together, exacerbate compromised PFC related to executive functions. If altered PFC has poor inhibitory control over the amygdalar reactivity, a vicious cycle can occur (1 ⇄ 8). Of note, besides environmentally mediated pathways, childhood BV can also be a risk factor if the maladaptive brain dynamics pre-date the stress. For instance, genetic factors and developmental nuances of greater amygdalar sensitivity to stress and/or immature functions of the PFC (e.g., poor ToM) can increase susceptibility to stress in early life. The cerebral networks potentially display hemispheric lateralization in functions that distinguish pure targets (the right dominance) from bully-victims (the left dominance). *Legend*: dACC, dorsal anterior cingulate cortex; BV, bullying victimization; ToM, theory of mind; ↑, increase; ↓, decrease.

Aversive stimulation/stress can activate (i.e., higher 5-HT release) the dorsal raphe nucleus (and consequently the amygdala and striatum) and reduce its inhibition *via* diminished input from the vmPFC (Maier et al., 2006; Christianson and Greenwood, 2014); whereas inhibitory control of the vmPFC over brainstem and limbic structures determines stressor controllability and resilience (e.g., Amat et al., 2005; Maier and Watkins, 2010). In analogy to mental resilience and coping with general psychological stress (Palamarchuk and Vaillancourt, 2021), we predict that the mental health issues associated with BV in childhood are related to an escalated perception of helplessness and loss of control (hypofunctioning of the dorsomedial PFC linked to hyperactivated midbrain raphe nuclei and altered serotonergic signaling) associated with being abused (hyperactivated amygdala and norepinephrine surge) and weaker executive functioning that results in poor behavioral control and depression. In other words, the PFC, which is responsible for emotional control of impulses and behavioral responses to stimuli (i.e., inhibitory control) is hijacked by the area of the brain responsible for more primal functions like survival (i.e., the limbic system, specifically the amygdala and insula) following fear conditioning (e.g., Etkin and Wager, 2007; Figures 1–3). *Vice versa*, poor executive functioning is also a risk factor for BV in young children. Preliminary results by Vargas et al. (2019) showed that in youth, increased exposure to BV was independently associated with lower volumes of the right medial orbitofrontal area (BA 11, which is a part of the vmPFC). As well, clinical high-risk youth, who displayed altered gray and white matter, were more exposed to BV compared to healthy youth. It may relate to the altered social skills and inhibition problems (i.e., impulsive behavior). In particular, Verlinden et al. (2014) demonstrated that the risk for being bullied in first and second grade was associated with inhibition problems at age 4, but did not relate to working memory, shifting (i.e., cognitive flexibility), planning/organization, or emotional control. Inhibition difficulties, for instance, can be seen in the amygdalar hyperactivity and greater positive functional connectivity between the amygdala and rostral ACC (i.e., social pain network) in children with attention-deficit/hyperactivity disorder (ADHD; Hulvershorn et al., 2014). This functional pattern of the social pain network is similar to the one found in bullied girls by Rudolph et al. (2016). This might explain why children with ADHD—who also have more academic, educational, and social problems—are at higher risk for BV than non-ADHD peers (Loe and Feldman, 2007; Holmberg and Hjern, 2008; Wiener and Mak, 2009; Murray-Close et al., 2010; Taylor et al., 2010; Efron et al., 2021).

#### Executive Functioning: Semantic Cognition

We hypothesize that the stress children experience because of being bullied by their peers will also present as poor inhibitory control that negatively impacts semantic performance, i.e., the coherence or correspondence in comprehension and abstract thinking, which consequently impairs memory and decision-making ability. This hypothesis is based on PTSD studies demonstrating that stress-associated cognitive deficits are linked to altered memory formation due to retroactive interference (Yehuda et al., 1995). Retroactive interference relates to higher cross-categorization, which refers to altered patterning discrimination by individual cues that are present in correct and incorrect responses. The effect of retroactive interference—when a new memory interferes with the retrieval of an old memory—is more pronounced in children than in adults because children attend to too many details and cannot discern complex patterns if they are missing details or extraneous cues (e.g., Darby and Sloutsky, 2015). Consistent with this idea, Vaillancourt et al. (2011) found that altered cortisol levels in bullied adolescents predicted neurocognitive dysfunction in visual and verbal learning, as well as in executive tasks that were related to learning strategies and decision-making.

During maturation, semantic cognition capacity increases with the development of the anterior (rostral) PFC (orbitofrontal cortex, BA 10; Dumontheil, 2014), which have been found to be positively associated with levels of social anxiety in childhood and adolescence (Rosso et al., 2004). At the same time, intense stress can induce suggestive pressure or spontaneous semantic priming (e.g., interpretation of innocuous events as threats), and thus facilitate retrieval of false memories (Payne et al., 2007; Otgaar et al., 2017). Although the tendency to falsely remember events increases during normal development in human cognition due to a paradoxical complementarity effect—an increase in both accurate and false recall/recognition with age (Metzger et al., 2008; Howe et al., 2009; Brainerd et al., 2018), there are opposite developmental trends in false memory due to deficient cognitive ability to form semantic relations (i.e., semantic cognition; Ralph et al., 2017) in young children (Brainerd and Reyna, 2007). That is, false memory formation decreases with age for semantically unrelated information but increases with age for semantically related information. Besides the increase in the strength of these associative links between pieces of information (i.e., backward associative strength), its automaticity also prevents inhibition (forgetting) of false memories with age (McDermott and Roediger, 1998; Gallo and Roediger, 2002; Howe, 2005). In children, false recognition can be promoted by increased rates of similarity that leads to a gist extraction from semantically related information (i.e., fuzzy face theory; Brainerd and Reyna, 2005), as well by false identity judgment about distractors or decreased rates of non-identity judgment (Brainerd and Reyna, 1998).

These developmental nuances of false memory formation and maintenance hypothetically interplay with the stress-induced executive dysfunctions in bullied children. This complex cognitive interplay can explain the discrepancies between retrospective accounts of BV in relation to actual self-reports of BV. For example, Nishina and Parra (2019) found that students who overreported their abuse by peers had poor current (12th grade) psychosocial adjustment (i.e., higher depressive symptoms and social anxiety, and lower self-worth). Although the authors did not identify the underpinning cognitive mechanisms, they referred to an analogy with a mood-congruent memory bias (i.e., induced by emotional valence of the content/stimuli; Miranda and Kihlstrom, 2005) for over-reporters. Such biases relate to a triggered negative mood by aversive content that can distort memory; yet over-reporters presented with “naturally” negative mood (e.g., depressive symptoms) that can endorse false recall (Bookbinder and Brainerd, 2016).

We relate these types of discrepancies to false memories due to compromised semantic ability associated with psychological maladjustment following severe stress perception in over- reporters. Cognitive appraisal has a strong moderating effect on emotional dysregulation and is thus linked to psychiatric outcomes such as mood and anxiety disorders (Picó-Pérez et al., 2017; Zilverstand et al., 2017). For instance, Graham et al. (2003) showed that BV identified by self-reports but not peer-reports predicted similar psychological maladjustments as did BV identified by both self- and peer-reports for 6th grade students. Furthermore, studies that isolate functional activity uniquely to false memories (e.g., hit baseline) have shown that false memory retrieval occurs within the network of cognitive control/appraisal and emotion regulation, that includes the vmPFC (BA 24) and dorsal anterior cingulate cortex (ACC, BA 34/24, 32), as well extending to the frontoparietooccipital regions (BA 6/44, 40, 18/19) and the brainstem; whereas activity in verbal processing share same regions with semantic false memories, which includes dorsomedial (BA 32/8, 6/8) and vlPFC (BA 45/47), dorsal ACC (BA 32), and frontoparietal regions (BA 6/44, 40/7; see a voxel-wise quantitative meta-analysis by Kurkela and Dennis, 2016). Thus, verbal memory and false memory processing share a network with cognitive appraisal, which can be compromised by high rates of self-perceived stress that is associated with psychological maladjustments. Accordingly, Slattery et al. (2013) demonstrated the impact of low cognitive appraisal on poor cortisol response to a social stress test in adolescents at age 18; whereby internalizing disorders increased the links between poor verbal memory performance and poor cortisol reactivity. Woody et al. (2018) found that social stress elicited cortisol and cardiovascular responses to the speech stressor; while there were no associations between cognitive load/intelligence and stress cortisol responses (Sladek et al., 2016; Woody et al., 2018). Lastly, executive dysfunction may also explain why youth who underreported being bullied had poorer 6th grade adjustment (Nishina and Parra, 2019), which cannot be supported by a mood-congruent memory bias. Rather, the under-reporting could be due to poor post-stress recall; especially since true memories undergo retrieval-induced forgetting, which is less applicable for false memories in children (Price and Phenix, 2015). Baugerud et al. (2016) demonstrated that maltreated children had a poor true recall for both neutral and emotional information, but higher rates of false recall for aversive information compared to their non-maltreated peers. Altered post-stress recall could be a maladaptive strategy to deal with the stressor, the interpretation of that stressor, and the memory of the stressor. This hypothesis resonates with the findings that in bullied children, depression is an independent contributing factor to memory deficit including verbal domain, which is negatively correlated to the PFC’s executive functioning in addition to elevated cortisol levels (Vaillancourt et al., 2011).

## Developmental Aspects and Interacting Neural Mechanisms in Childhood Stress Psychopathology

### Sensitivity to Facial Expressions

Fear-conditioned memories of faces can impair the fusiform gyrus (Mueller and Pizzagalli, 2015), which functional significance is (but not limited to) in facial expression perception and face recognition (Kanwisher and Yovel, 2006; Iidaka, 2014). A recent study showed that high BV at age 8 years was associated with a “thicker cortex” of the fusiform gyrus in high-resolution structural magnetic resonance imaging (MRI) at age 10 years (Muetzel et al., 2019). Although true neural basis is unclear, in pediatric neuroimages, what appears to be larger cortical thickness, could in fact be due to delayed myelination. Myelination, the axonal insulation that facilitates signaling (action potentials) within synapses, is an essential neuronal networks component which undergoes critical developmental changes across ages 5–10 years. The increase in myelin deposition alters the contrast between gray (cortex) and white matter (which mainly contains long-range myelinated axons); thus, cerebral maturation can appear as cortical thinning after reaching peak by age ~10 years (Shaw et al., 2008; Ducharme et al., 2016; Natu et al., 2019). The reported greater thickness of the left fusiform gyrus (Muetzel et al., 2019) may in fact be related to delayed myelination in bullied children. However, more research is needed to assess the impact of potential mediators and moderators. For example, socioeconomic status was found to moderate trajectories of the cortical thickness decline, which had a curvilinear pattern (relatively steep decline in early childhood and subsequent leveling off during adolescence) for the low levels and linear pattern for the high levels, with the major effect in left superior temporal gyrus (Piccolo et al., 2016). As well, ADHD is associated with delayed maturation of the PFC (Shaw et al., 2007), whereas ADHD outcome mediates cortical thickness—the worse outcome is related to a “fixed” thinning of the left medial PFC at baseline (mean age 8.9 years) and the better outcome is related to the normalization of cortical thickness in the right parietal cortex at follow-up (mean age 5.7 years; Shaw et al., 2006).

Cortical thickness is an age-related parameter in structural MRI studies; however, the fact is that cortical thinning can relate to various factors of neurogenesis (e.g., synaptic reorganization and pruning; Shaw et al., 2008). As well, we see the interpretation during “thickening → thinning” transitioning period at age 10 years is especially challenging; conversely, longitudinal studies that define trajectories of cortical development and cortical thickness reached peaks would provide more insights into the underpinning cellular mechanisms. It cannot be explained solely by structural MRI and *in vivo*; an integration of other parameters is needed such as cortical pattern matching to account for gray matter variation and gyral patterning (Shaw et al., 2008; Tamnes et al., 2010). Maturation is also associated with functional connectivity transformations when cortical control within cerebral networks enhances and other cerebral regions, including the fusiform gyrus, lose centrality during the transition to adolescence period (Sato et al., 2015). Thus, we relate the reported greater cortical thickness (Muetzel et al., 2019) to delayed maturation that can be clarified with the cortical development trajectories in bullied children. The greater cortical thickness can also be a risk factor for cerebral functional connectivity due to local white matter alterations, which has to be further tested with functional MRI and fractional anisotropy.

Although Muetzel et al. (2019) do not account for the aforementioned neuroimaging specifics, that is, the delayed myelination rather than cortical hypertrophy, they did discuss that these cortical morphology changes in the fusiform gyrus could be related to a sensitivity to aggressive facial expressions developed in BV targets. Indeed, nonverbal facial expressions recognition involves emotional contagion (part of affective empathy) *via* brain-to-brain coupling and mimicry between individuals’ emotion systems (Prochazkova and Kret, 2017), whereas the amygdala can increase the sensitivity of the fusiform gyrus to visual response to static fearful faces accordingly to the connectivity models of fear sensitivity by Furl et al. (2013). However, we predict that this stress mechanism likely extends beyond this type of amplified emotional response to fearful/aggressive faces and involves functional connectivity (white matter architecture) that alters the fusiform gyrus input into theory-of-mind (ToM) *via* developmental sensitization in BV.

### Theory of Mind

Theory of Mind (ToM), also known as mentalization, refers to the ability to infer the mental states of others like their thoughts/beliefs (i.e., cognitive perspective-taking or cognitive ToM) and emotions/feelings (i.e., affective perspective-taking or affective ToM) and the analysis of related behavior that is essential in social interactions and problem-solving (Dvash and Shamay-Tsoory, 2014; Healey and Grossman, 2018). Both cognitive and affective ToM engage the temporoparietal junction, precuneus, and temporal poles. Yet, cognitive ToM may engage the dorsomedial and dorsolateral PFC; whereas affective ToM may uniquely engage the vmPFC, the amygdala, and basal ganglia (that includes striatum; Healey and Grossman, 2018). The fusiform gyrus facilitates understanding intentions during ToM processing *via* the visual perception of others’ emotions (i.e., “reading” eye expressions or body language; Schurz et al., 2013, 2014; Thye et al., 2018; Tallarita et al., 2020). During social interactions, face processing relates to the activation of the fusiform gyrus, which is significantly influenced by emotional input from the amygdala; the latter produces fast perceptions of social–emotional feelings that are moderated by previous social experiences (i.e., subconscious threat-monitoring; Schultz et al., 2003; Duncan and Barrett, 2007; Herrington et al., 2011; Balderston et al., 2014; Frank et al., 2019). The amygdala’s hyperactivity may therefore disturb networks with the fusiform gyrus and compromise ToM processing (insufficient input from the vmPFC), which can be seen in rigid habit-like behavior during stressful social interactions due to the limbic dysregulation. In fact, Frick et al. (2013) showed that increased sensitivity of the fusiform gyrus to fearful faces was accompanied by the changes in connectivity between the fusiform gyrus and amygdala (increased) and also between the fusiform gyrus and vmPFC (decreased) in social anxiety disorder. Rudolph et al. (2016) also showed that the fusiform gyrus over responded to exclusion (relative to inclusion) together with the amygdala and dorsal ACC, the brain structures which hyper responses consequently predicted internalizing symptoms in bullied girls. In contrast, conscious awareness (the dorsomedial PFC activation in emotional downregulation) of the fearful faces can reduce the amygdala-PFC functional connectivity; whereas the connectivity is increased when information is processed non-consciously and the amygdalar responses relate to a negative bias in the subsequent evaluation of neutral faces (Lapate et al., 2016). That is, cognitive behavioral therapy that facilitates proper evaluation of the fearful faces (i.e., cognitive reappraisal) may help with overcoming explicit biases and subconsciously/“automatically” triggered social anxiety.

The need to support cognitive re-appraisal may also relate to poor executive functions in BV. Du Plessis et al. (2019) showed that childhood BV was related to a structure of the right vlPFC (i.e., thickness and surface area, moderated by cortisol levels), yet only in adolescent boys. *Vice versa*, stress-associated impairment of ToM likely can promote attentional tunneling that reduces cognitive flexibility (i.e., poor functioning of the medial/ventral PFC affects the dorsal/lateral PFC activity), and in turn, contribute to academic underperformance of bullied children. Clemmensen et al. (2020) showed that ToM and involvement in BV were both independently associated with academic performance in children aged 11–12 years, even after accounting for IQ and shared variance. Moreover, in this study, bullied girls had lower academic performance than non-bullied girls and boys and bullied boys.

### Emotion Regulation: Prefrontal Cortical Descending Projections

The neurobiological background of ToM is that the medial PFC is critical for social cognition like judgment. Although the vmPFC is linked to understanding affective but not cognitive ToM (Shamay-Tsoory et al., 2006; Shamay-Tsoory and Aharon-Peretz, 2007), it is an anatomical key element for social, affective, and cognitive networks; and its disruption is linked to psychopathology and psychiatry (Hiser and Koenigs, 2018). Our hypothesis is that the complex interplay between altered vmPFC’s circuits linked to ToM affects the amygdalar responses to aversive emotions and fear, which relates to stress sensitization and psychiatric outcomes in bullied children.

First, the vmPFC is a reward-sensitive region that promotes social connection to others, monitors safety, and inhibits emotional-related reactions (Delgado et al., 2006). The vmPFC and left amygdala coactivity (i.e., concurrent responses during emotion processing) occurs during active regulation of negative emotions (Diekhof et al., 2011; Yang et al., 2020). The evidence for adults is that negative coactivation between the vmPFC (i.e., hyperactivity) and right amygdala (i.e., hypoactivity) influences downregulation of negative emotions, which is a cognitive “minimizing” reappraisal (Banks et al., 2007; Morawetz et al., 2017; Yang et al., 2020). Specifically, the vmPFC encodes emotional value (e.g., perceived aversiveness and fear) and further modulates it during cognitive reappraisal (e.g., distancing from feelings or reward anticipation). The successful emotion downregulation concordantly hypoactivates the left amygdala (i.e., negative coactivation that suppresses emotional value) and extends to the nucleus accumbens (the ventral striatum) to suppress reward encoding (Phan et al., 2005; Staudinger et al., 2009; Mulej Bratec et al., 2017). Thereby, we may say that successful emotional regulation relates to the pattern of top-down (i.e., cortical → subcortical) negative coactivity: the vmPFC hyperactivity → the right amygdalar hypoactivity (then mirrored by the left amygdala) → the ventral striatum hypoactivity. Of note, the cerebral activity pattern differs during the cognitive “positive” reappraisal (i.e., finding the positive meaning in negative experiences) as displays positive coactivity in this functional architecture (Doré et al., 2017), which likely contributed to Dougherty et al.’s (2015) findings of the “divergent” effect in children reappraisal (see also“self-appraisal” vs. “stressor-appraisal”; Palamarchuk and Vaillancourt, 2021).

However, BV is associated with emotional dysregulation related to the PFC dysfunction. In bullied young adults, the medial PFC-amygdalar connectivity across inclusion and exclusion was found to be positive (i.e., the medial PFC hyperactivity → the right amygdalar hyperactivity; see Figure 3) and indicated insufficient inhibitory control over the amygdala; this positive connectivity also moderated the relation between BV and depressive symptoms (McIver et al., 2019). For bullied adolescent girls, Casement et al. (2014) showed that the medial PFC and striatal hyper responses to reward anticipation mediated the link between social stressors and concurrent symptoms of depression; while Telzer et al. (2020) found that severity of BV correlated to hyperactivity in the amygdala and ventral striatum (but not limited to these structures). In bullied adolescents, it was shown that emotion regulation moderated poor cortisol reactivity to stress (Kliewer, 2016); as well, emotion regulation was associated with the emotion awareness and later on with anger regulation and lower rates of BV during the 2-years follow-up (Riley et al., 2019). In young children, Park et al. (2021) showed that chronic stress exposure can affect resting-state functional connectivity: (1) decrease it between the medial PFC and ventral tegmental area, which is essential in reward cognition such as associative learning and motivational salience; yet (2) increase it between the anterior hippocampus and left inferior frontal gyrus (BA 44), which is a part of the vlPFC implicated in language production and phonological/speech processing. These brain dynamics support the aforementioned findings that chronic BV can impair academic performance and increase the vulnerability of young targets. Similarly, mood and anxiety disorders were found to relate to less successful emotional downregulation in cognitive reappraisal (i.e., distancing strategies related to the vmPFC; Picó-Pérez et al., 2017), while the amygdalar hyper responses were linked to depression (Zilverstand et al., 2017). That is, emotional dysregulation in bullied children is likely linked to poor ToM that compromises cognitive appraisal and exhibits the pattern of top-down positive coactivity the vmPFC hyperactivity → the amygdalar hyperactivity (Figure 3).

Second, a successful “minimizing” reappraisal circuit also involves the positive coactivation of the social pain network, the vmPFC, the ACC, and the insula (Diekhof et al., 2011; Winecoff et al., 2013; Doré et al., 2017). For instance, Phan et al. (2005) showed that increases in negative emotional experiences in healthy adults were related to hyperactivity in the amygdala and hypoactivity in the dorsal ACC, which was moderated (attenuated and augmented, respectively) during cognitive reappraisal that involved activation of the dorsomedial PFC. The ACC is a behavior-monitoring and optimal decision-making region that integrates contextual socio-emotional information such as perceived fairness/unfairness (e.g., Kennerley et al., 2006; Lavin et al., 2013; Rolls, 2019). Activity in the dorsal/rostral ACC for fearful faces is negatively associated with BV (Swartz et al., 2019). Of note, a rostral part of dorsal ACC (often termed as the rostral ACC) belongs to the vmPFC (BA 32). Changes in functional coactivity within the social pain network (Figure 3) may explain heightened rejection/interpersonal sensitivity in targets compared to perpetrators; that has been shown to mediate the association between BV and mental health symptoms (Williams et al., 2017; McDonnell et al., 2018). The hypothesis resonates with Kross et al.’s (2007) findings that self-reported ratings of rejection-associated distress negatively correlated with hyperactivity in the left dorsal ACC (BA 6, which activity correlated positively with the right dorsomedial PFC, BA 8) and right insula in young healthy adults. As well, compared to high rejection sensitivity, low rejection sensitivity was associated with hyperactivity in the lateral PFC (left BA 45, 9, and right BA 6). Baird et al. (2010) demonstrated that increases in the dorsal ACC (anterior, BA 24/32 and posterior, BA 31) and bilateral dorsolateral PFC coactivity was associated with reduced sensitivity to relational aggression during an affect recognition task in bullied adolescent girls. This denotes a moderating role of the executive functions in emotion regulation. McIver et al. (2019) showed that social exclusion (compared to inclusion) increased the ACC-right insula functional connectivity in bullied and non-bullied young adults. However, positive connectivity between the left amygdala-ACC and left amygdala-right insula were attenuated in bullied adults, compared to non-bullied adults. That is, emotional dysregulation in bullied children is likely moderated by heightened rejection sensitivity with the pattern of top-down coactivity lateralized to the right: the vmPFC hyperactivity (i.e., poor affective ToM) → the dorsal ACC hypoactivity (higher rejection sensitivity) → the amygdalar hyperactivity → the right insula hyperactivity (Figure 3).

The dorsal ACC over responses to social exclusion in both BV and chronic peer rejection (Rudolph et al., 2016; Will et al., 2016), and its coactivation with anterior insula during social exclusion predicts increased internalizing symptoms and avoidance motivation in bullied girls (Rudolph et al., 2016). However, the reactivity of the left insula, as well as of the left nucleus accumbens (ventral striatum) appears to be additionally moderated by both adolescent depression (Silk et al., 2014) and bullying behavior (Perino et al., 2019). Importantly, executive functioning capacity also modulates the association between the activity in the left insula and dorsal ACC linked to aggression: low executive functioning predicts positive association and high executive functioning predicts negative association (Chester et al., 2014); whereas the dorsal ACC-insula functional connectivity correlates to target-changing behavior, which is linked to extraversion and thus, hypothetically, could facilitate increased interpersonal aggression (Takami and Haruno, 2020).

Perino et al. (2019) demonstrated that adolescents’ self-reported BV was related to hyperactivation in the medial PFC, insula, amygdala, and ventral striatum; yet Beekman et al. (2016) revealed that the need to belong can moderate the stress-responses to social exclusion. Chester et al. (2016) showed that rejection, as compared to acceptance, was not directly associated with a greater need to belong, but it was indirectly *via* social pain. Specifically, higher activity in the dorsal ACC and the insula predicted affiliative behavior. Thus, specifics of the dorsal ACC-insula functional coactivity, which are moderated by both executive dysfunctioning and rejection sensitivity, may differ between pure targets and bully-victims (i.e., children who are bullied and bully others; Figure 3); and because bullied children can have an anxious need to belong, which may be why they become perpetrators (Barker et al., 2008; Haltigan and Vaillancourt, 2014; Underwood and Ehrenreich, 2014). The poor outcomes for BV targets might thus be due to a socioemotional asymmetry that is characterized by a higher sensitivity to rejection vs. the higher need to belong seen in perpetrators. Perpetrators of bullying have been shown to have status goals related to dominance and prestige (Sijtsema et al., 2009) and often enjoy greater power, dominance, and popularity than BV targets (Vaillancourt et al., 2003; Pouwels et al., 2018; Faris et al., 2020).

As well, bullying behavior may relate to greater impulsivity (related to cognitive inflexibility associated with the dorsal/lateral PFC, reviewed above), which was found to be associated with the cortical volume reductions (in the medial PFC and insula) but subcortical volume increases (in the ventral striatum, hypothalamus, and anterior thalamus), whereas cortical to subcortical volume ratio partially mediated the association between early adversity and antisocial behavior (Mackey et al., 2017). That is, in bully-victims, the dorsal ACC—left insula coactivity likely has stronger functional connectivity and relates to an increased need to belong, aggression, and impulsivity but lesser rejection sensitivity compared to pure targets (Figure 3).

The dorsal ACC-insula effects on the amygdalar responses related to impulsivity and rejection sensitivity that distinguishes pure targets from bully-victims may belong to their empathic ability; and their neural network is also regulated by these structures (Völlm et al., 2006; Decety et al., 2013a, b). Empathic ability is an emotional aspect of inferring and sharing the emotional experiences of others (Dvash and Shamay-Tsoory, 2014), which entitles affective empathy (i.e., affective ToM, the vmPFC) and emotional contagion (Healey and Grossman, 2018). During social interactions, empathy levels mediate spontaneous engagement of cognitive ToM (i.e., cognitive empathy) related to the dorsomedial PFC (Pluta et al., 2011; Wagner et al., 2011). Its deterioration in the right hemisphere is associated with not understanding others’ emotions (Ratka, 2018). In mentalizing, the activation of the dorsomedial PFC is less pronounced in relation to negative social stimuli like social exclusion compared to more positive ones like social inclusion (Powers et al., 2013). This is likely an adaptive tool, a type of self-defensive mechanism that involves mental withdrawal to numb emotional pain. In fact, social exclusion is related to a higher tolerance for physical pain and “emotional numbness” such as reduced joy and empathy, which are intercorrelated (DeWall and Baumeister, 2006; Powers et al., 2013). Accordingly, we hypothesize that the aforementioned poorer ToM ability in children who are bully-victims compared to pure targets and pure perpetrators (Shakoor et al., 2012) is related to the vmPFC functioning being moderated by the dorsomedial and dorsolateral PFC activity (i.e., cognitive empathy and executive functioning; see Figure 3). This may also explain the findings that depression, anxiety, and psychosomatic symptoms were most frequently observed in bully-victims, but equally observed in pure targets and pure perpetrators (Kaltiala-Heino et al., 2000).

### Behavioral Modulation: Amygdalar Ascending Projections

Meta-analytic findings of the emotional processing of fear in individuals with PTSD, social anxiety disorder, and specific phobia reveal hyperactivity in the amygdala and insula compared to healthy participants undergoing fear conditioning. Of note, the patterns of coactivation between the cortical regions of interest were observed only in individuals with PTSD (Etkin and Wager, 2007). Specifically, the patterns included: (1) negative coactivation between the right amygdala (its hyperactivity was then mirrored by left amygdala) and right dorsal/rostral ACC (its hypoactivity was then mirrored by the left cortex); and (2) positive coactivation between the latter cortex and the right (and then left) vmPFC. Conversely, extinction of conditioned fear relates to hypoactivity in the amygdala and hyperactivity in the dorsal/rostral ACC and the vmPFC (Phelps et al., 2004). Considering the amygdala-PFC connectivity, the main pathway for behavioral modulation in bullied children hypothetically can be: fear conditioning → the right amygdala hyperactivity (then mirrored by left amygdala) → hypoactivated dorsal/rostral ACC → hypoactivated vmPFC → poor affective ToM, higher rejection sensitivity, and higher distress (Figure 3).

Phobia and distress can have a larger effect on the PFC in childhood than in adulthood. ToM ability is facilitated by the inferior temporal lobe extending into the fusiform gyrus, which emerges around the ages of 3–5 years and is solidified in most children by around age 7 (Sabbagh et al., 2009; Lackner et al., 2010). Yet, ToM reasoning activity in the medial PFC shifts from ventral to dorsal only in late childhood (Moriguchi et al., 2007) and the affective component of empathy develops earlier than the cognitive component (Decety and Svetlova, 2012). The neuroanatomical background is that ToM development relates to the white matter maturation in: (1) “local structure” of (near) the medial PFC, medial parietal (precuneus), and temporoparietal regions, and (2) connectivity between the inferior frontal and temporoparietal regions (Grosse Wiesmann et al., 2017); whereas white matter tracts of the frontotemporal regions have a pattern of late maturation (Tamnes et al., 2010). The cortical functional connectivity development is also asymmetrical—earlier in the right circuits and more expressed in the anterior (dorsal frontoparietal) regions (Sowell et al., 2004; Zhu et al., 2011).

Thus, the medial PFC (i.e., anterior region) is largely influenced by the right amygdala (i.e., subcortical region). However, the medial PFC is neither capable to execute sufficient inhibitory control over the amygdala during aversive stimuli nor is supported enough by the dorsal PFC related to cognitive reasoning due to its late maturation. Not surprisingly, the pre adolescent period is a high-risk time for stress to alter the maturation and functioning of the PFC (Caballero et al., 2016). This explains why the vmPFC (especially in the right hemisphere) is susceptible to behavioral modulation imposed by the amygdala’s hyperactivation in fear conditioning/emotional face perception, which is greater in children than in adults (Monk et al., 2003; Guyer et al., 2008), and linked to psychopathology, such as major depression and bipolar disorder (Monk et al., 2008; Brotman et al., 2014; Wegbreit et al., 2014).

Barker et al. (2008) and Haltigan and Vaillancourt (2014) examined the joint trajectories of BV and perpetration across childhood and adolescence respectively and found that the evolution of bullying involvement emerged from victimization to perpetration and not the reverse. A meta-analysis by Walters (2021) further revealed that BV and perpetration are intercorrelated and have bidirectional cross-lagged longitudinal relations, i.e., perpetration ⇄ victimization. High BV seems to exacerbate poor affective reasoning and emotional control. Indeed, higher levels of BV in children are related to lower conscientiousness and higher neuroticism, which manifests as feeling angrier, blaming the perpetrator, and being less forgiving during conflicts (Bollmer et al., 2006), which could indicate ToM nuances linked to asymmetry in the PFC’s control over the amygdalar responses to anger and fear that distinguish pure targets from bully-victims. In particular, poor ToM at age 5 years was found to be related to higher levels of reactive aggression (i.e., limbic/automatic response to a real/perceived threat) at age 6 years in BV targets; whereas better ToM at age 5 years was found to be related to higher levels of proactive aggression (i.e., a non-provoked/planned behavior for personal gain) at age 6 years in perpetrators (Renouf et al., 2010). As well, the amygdala’s higher response to anger and lower response to fear predicts bullying behavior (Swartz et al., 2019). Regardless of the motivational direction (i.e., approach vs. withdrawal), higher levels of anger have been related to larger left lateralization in the PFC activity (Wacker et al., 2003; Stewart et al., 2008), while the right amygdalar hyperactivity is a frontline response to fear conditioning linked to hypoactivity of the right medial PFC (Etkin and Wager, 2007), and the latter correlates to impaired detection of deception (Stuss et al., 2001; Beekman et al., 2016). Moreover, emotional valence asymmetry in cerebral functioning is well-documented (Davidson, 1992); and the hypoactivity in the right cortex relates to poor recognizing of negative emotion such as envy, whereas hypoactivity in the left cortex relates to poor recognition of positive emotion (Shamay-Tsoory et al., 2007). We hypothesize that poor ToM is linked to the amygdala-medial PFC coactivity, which is moderated mainly by fear, and in the right hemisphere in pure targets compared to bully-victims, whose functional coactivity is moderated mainly by anger and in the left hemisphere (Figure 3).

## Discussion: Stress Vulnerability Nuances and Integrative Brain Dynamics

BV is associated with a significant psychological burden that triggers a neuroendocrine cascade of stress-response activated by elevated cortisol levels and the outcomes are seen in the long-lasting deterioration of mental health, including depression, PTSD, and suicidal ideations in children and adolescents (Ttofi et al., 2011; Moore et al., 2017; Vaillancourt, 2018; Vaillancourt and Palamarchuk, 2021). BV can develop into an interpersonal trauma (Idsoe et al., 2021) where psychopathology is a by-product of a multilevel neural phenomenon with the brain dynamics bidirectionally stemming from the cortical-subcortical cerebral circuits. Given the scarcity of research that can clarify the cognitive and emotional convergence underlying psychopathology associated with childhood BV, we review and integrate the evidence on brain dynamics in: (A) general psychological stress; (B) childhood BV; and (C) early life adversity (see schematic outlining of the major cerebral circuits in Figure 3), and in doing so, emphasize the following aspects:

(A). The integral role in cognitive and emotional convergence belongs to the amygdala functioning and its circuits triggered by fear in acute stress. Fear conditioning can lead to psychiatric problems because the amygdala is the emotional hub of memory and influences the cognitive “defence” mechanism on a subconscious level, which is represented in the vm fronto-temporoparietal network (e.g., Palamarchuk and Vaillancourt, 2021).

(B). Perspective-taking abilities (i.e., ToM) play an essential role in social interactions. Both cognitive and affective ToM share the vm fronto-temporoparietal network with cognitive “defence” mechanism. Immature and/or stress-compromised ToM relates to the misperception of others’ intentions and can contribute to explicit bias and anxiety (reviewed above). This can narrow attention with a feeling of losing control over the situation. At the same time, cognitive inflexibility and insufficiency in emotion regulation aggravates stress perception and worsens psychological disturbance (e.g., Palamarchuk and Vaillancourt, 2021). Not surprisingly then, BV is linked to poor ToM.

(C). From the early life stress viewpoint, there are several major nuances that can explain why BV is reliably associated with a risk of developing psychiatric disorders in childhood. First, the higher risk of psychiatric disorders in bullied children is likely supported by developmental aspects in the anatomical ToM signature besides emotion reactivity. In particular, the higher amygdalar response to fear (seen in a norepinephrine surge) and its stronger influence on the vmPFC (seen in the negative coactivity of these brain structures) in children compared to adults could increase the risk of disorders. Second, the disturbed emotional processing due to poor inhibitory control of the vmPFC can aggravate the amygdalar hyperactivity in stress. Third, stress-induced morphological and neurochemical changes within the cerebral networks are especially harmful in early life because of undergoing neurogenesis and myelination, which is likely compromised in young targets. Lastly, the susceptibility to psychiatric dysfunctioning among bullied children and adolescents can relate to the neuroepigenetics that moderate stress reactivity (e.g., blunted cortisol response).

Although we refer to the neural mechanisms of early life and general psychological stressors, the neuropathology in childhood BV may differ and longitudinal studies are required to test the brain dynamics models—integrative vs. specific—to better understand how BV gets under the skin to confer risk in young targets. Nevertheless, psychopathology in mental stress significantly relates to the cognitive appraisal of a stressor but not to the stressor *per se*; while the influencing factors are stressor’s acuity, timing, and novelty (e.g., unpredicted/novel vs. homotypic, e.g., for details see review by Palamarchuk and Vaillancourt, 2021). We thus hypothesize that the brain dynamics associated with childhood stress psychopathology depend on a stress perception/comprehension to a larger extent, compared with a type of victimization (e.g., child maltreatment vs. peer victimization). The integrative brain dynamics for BV and early life adversity potentially would not be significantly distinct since many of the associations are extrapolated from research on childhood stress in the broader sense; and oftentimes, BV does not exist in a “vacuum”. Although poly victimization is out of this review scope, we would suggest that children and youth who experience multiple types of victimization across different domains (child maltreatment, community violence, physical/sex abuse, etc.) are likely to be among those with the worst neurocognitive and psychiatric outcomes due to a complex stress response.

The goal of this review is to help inform future studies that are focused on the treatment and prevention of psychiatric disorders and learning problems in bullied children and adolescents. To move the research forward in this area, we offer the following recommendations. First, research on BV, as well as on any psychological stress, must identify the exact stressor(s), perceived stress severity, and perceived controllability of the stressor. Second, psychological/clinical symptoms must be well differentiated in BV target’s anamnesis (e.g., occurred prior to a stressor/event, induced by a specific stressor, random/transient/chronic, and aggravating/relieving factors). Third, studies on cognitive re-appraisal aimed to define neural correlates in emotion regulation must consider different effects of *minimizing appraisal* vs. *positive appraisal* and *self-appraisal* vs. *stressor-appraisal* (Palamarchuk and Vaillancourt, 2021).

Our hope is that the proposed neurobiological mechanisms can be used in the prediction of risk for neurocognitive vulnerability and psychopathology following BV in childhood. For instance, consideration of dopamine-related behavioral maladjustments may facilitate proper sociomedical interventions focused on preventing children from becoming the target of BV or bully-victim. As well, a better understanding of the association between BV and academic achievement can help young BV targets with expedited sociopsychological help, including cognitive behavioral therapy focused on associative learning, cognitive flexibility, and reappraisal to develop a more appropriate reactivity to stressors and successfully cope with the stress.

## Conclusion

BV is a psychological stress which can lead to pervasive interpersonal trauma that devastates far too many children worldwide. Being the target of BV places children at risk for a host of problems, most notably in the areas of mental health and learning. In our review, we delineate how exposure to this type of social pain is associated with a series of neurobiological responses that could result in psychopathology, as well as enduring structural and functional changes in the brain. We describe the complex cognitive and emotional convergence and synthesize the data in a form of integrative brain dynamics, as well as provide testable hypotheses to explain why children are more vulnerable to the adverse outcomes of BV than adults. Given how high the stakes are, the reduction of bullying must be prioritized.

## Author Contributions

TV encouraged and supported ISP to investigate the impact of psychological stress on cognition in bullied children. ISP planned and carried out the project, the main conceptual ideas, developed hypotheses, and integrative model of the brain dynamics associated with bullying victimization. ISP designed the figures and wrote the manuscript with notable input from TV. Both TV and ISP provided critical feedback, helped shape the manuscript, and contributed to its final version. All authors discussed the results and agreed to be accountable for the content of the work. All authors contributed to the article and approved the submitted version.

## Conflict of Interest

The authors declare that the research was conducted in the absence of any commercial or financial relationships that could be construed as a potential conflict of interest.

## Publisher’s Note

All claims expressed in this article are solely those of the authors and do not necessarily represent those of their affiliated organizations, or those of the publisher, the editors and the reviewers. Any product that may be evaluated in this article, or claim that may be made by its manufacturer, is not guaranteed or endorsed by the publisher.

## Abbreviations

ACC: anterior cingulate cortex
BA: Brodmann area
BV: bullying victimization
HPA: hypothalamic–pituitary–adrenal
PFC: prefrontal cortexprefrontal cortex
vl: ventrolateral
vm: ventromedial.
